# Bullet embolization to the external iliac artery after gunshot injury to the abdominal aorta: a case report

**DOI:** 10.1186/1752-1947-5-354

**Published:** 2011-08-05

**Authors:** Luan Jaha, Bekim Ademi, Vlora Ismaili-Jaha, Tatjana Andreevska

**Affiliations:** 1 Department of Vascular Surgery, University Clinical Center of Kosova, Rrethi i Spitalit pn, 10000 Prishtina, Republic of Kosova; 2 Department of Pediatrics, University Clinical Center of Kosova, Rrethi i Spitalit pn, 10000 Prishtina, Republic of Kosova; 3 Department of Thoracovascular Surgery, University "Kiril and Metodij", Skopje, Former Yugoslav Republic of Macedonia

## Abstract

**Introduction:**

Abdominal vascular trauma is fairly common in modern civilian life and is a highly lethal injury. However, if the projectile is small enough, if its energy is diminished when passing through the tissue and if the arterial system is elastic enough, the entry wound into the artery may close without exsanguination and therefore may not be fatal. A projectile captured may even travel downstream until it is arrested by the smaller distal vasculature. The occurrence of this phenomenon is rare and was first described by Trimble in 1968.

**Case presentation:**

Here we present a case of a 29-year-old Albanian man who, due to a gunshot injury to the back, suffered fracture of his twelfth thoracic and first lumbar vertebra, injury to the posterior wall of his abdominal aorta and then bullet embolism to his left external iliac artery. It is interesting that the signs of distal ischemia developed several hours after the exploratory surgery, raising the possibility that the bullet migrated in the interim or that there was a failure to recognize it during the exploratory surgery.

**Conclusion:**

In all cases where there is a gunshot injury to the abdomen or chest without an exit wound and with no projectile in the area, there should be a high index of suspicion for possible bullet embolism, particularly in the presence of the distal ischemia.

## Introduction

Abdominal vascular trauma is fairly common in modern civilian life and is a highly lethal injury, with overall mortality around 40% in some reported series. The main cause for this high mortality relates to problems transporting injured patients to the hospital fast enough to prevent exsanguination. Furthermore, abdominal vascular injuries are rarely isolated, and other organs are often severely damaged as well.

However, bullet penetration of the aorta is not always fatal. If the projectile is small enough and the arterial system elastic enough, the entry wound into the arterial channel may close without exsanguination. A small projectile thus captured will travel within the lumen with the current of blood flow until it is swept far enough to be halted by the diminishing diameter of tile peripheral vasculature. The occurrence of this phenomenon is rare. In 1968, Trimble [1] was the first to summarize the cases published until that time. There were 33 reports, dating back to 1885. He added two additional cases. Two more were added by Cyrus and Klein in 1972 [2], and since then there have been several others [3-14]. In these reports different techniques for treatment were presented, starting with very common methods to ones employing laparoscopic and endovascular techniques.

Here we present our experience with a gunshot injury through the lumbar vertebra to the posterior wall of the abdominal aorta, followed by bullet embolism to the left external iliac artery.

## Case report

**Figure 1:**
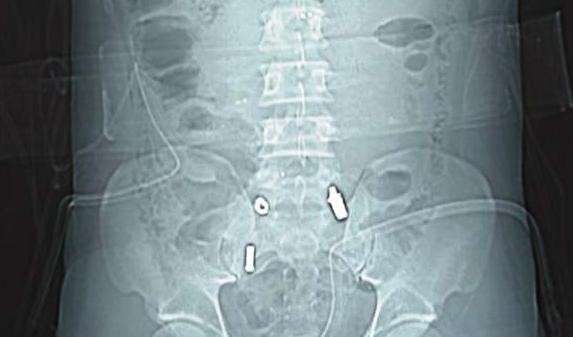
**CT scan of our patient with bullets**.

**Figure 2:**
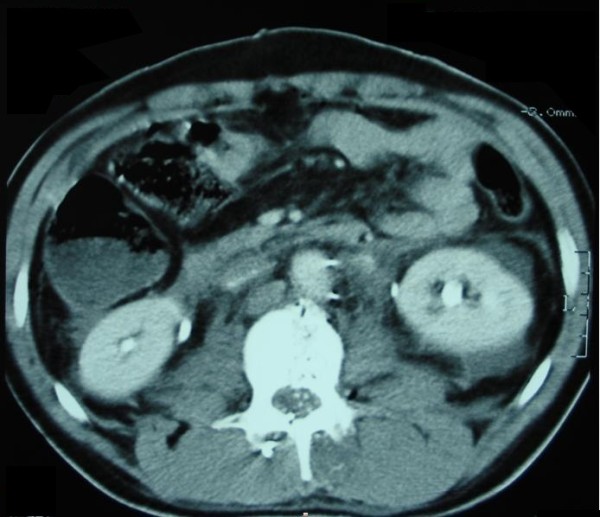
**Fracture of his twelfth thoracic vertebra**.

Three hours after being shot in the back, a 29-year-old Albanian man was admitted to the Surgical Department of our Emergency Center. An examination revealed two small caliber bullet holes over his thoracolumbar spine and sacrum, paraplegia and absence of the pulses. The deteriorating condition of our patient led to the decision to surgically explore his abdomen. No injuries to the viscera were found. A small retroperitoneal hematoma on his right side was opened. His pulse over his common iliac arteries was normal and there was no active bleeding at the area. Drains were placed and his abdomen was closed in layers. Because of an insufficient improvement of the monitored parameters, our patient was intubated and transferred to our intensive care unit for further resuscitation. Three hours later he developed ischemia in his left leg. His leg was cold, with no pulse up to the common femoral artery and there were signs of discoloration. Computed tomography of his chest and abdomen revealed two bullets - one in his left iliac fossa and a second in front of his sacrum (Figure 1). Color Doppler imaging revealed an obstruction of the external iliac artery on his left side. No free fluid was found in his abdomen. There was also a multiple fracture of his twelfth thoracic and first lumbar vertebrae with no free fluids in his abdomen (Figure 2). These findings alerted the vascular surgery team and after a consultation, a tentative diagnosis of a gunshot injury was made. The decision was made to re-enter the abdomen.

**Figure 3:**
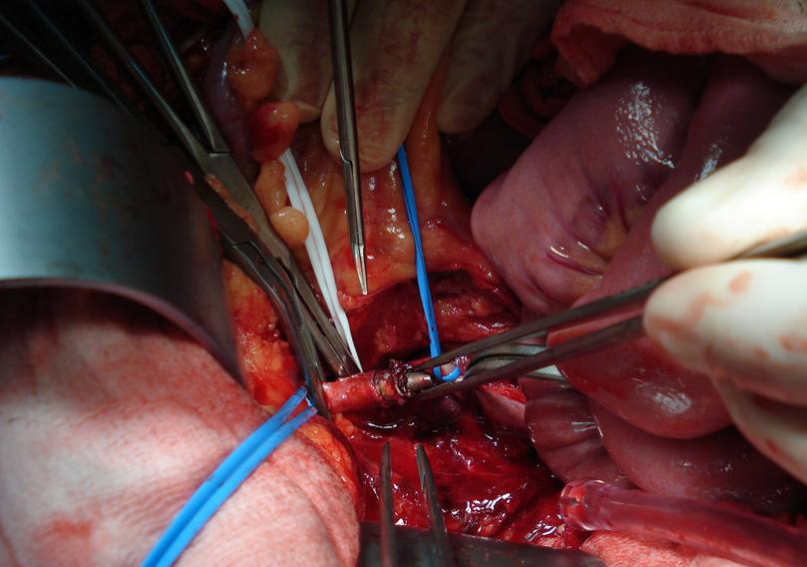
**Extraction of the bullet from the left common iliac artery**.

**Figure 4:**
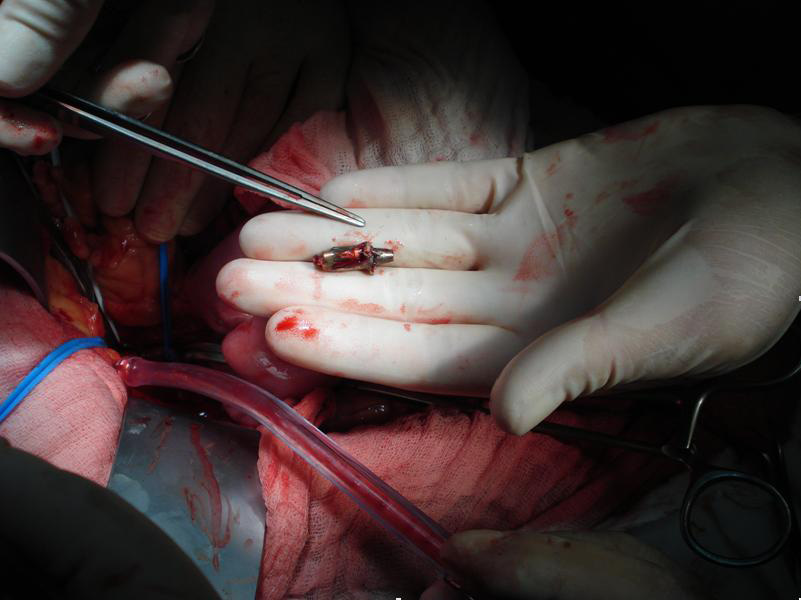
**Extracted bullet**.

**Figure 5:**
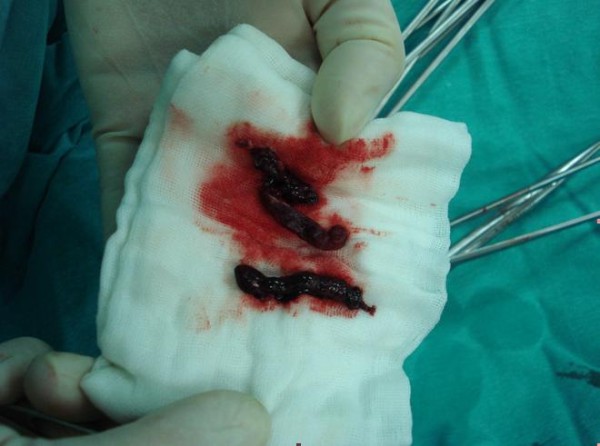
**Thrombectomized distal arteries**.

**Figure 6:**
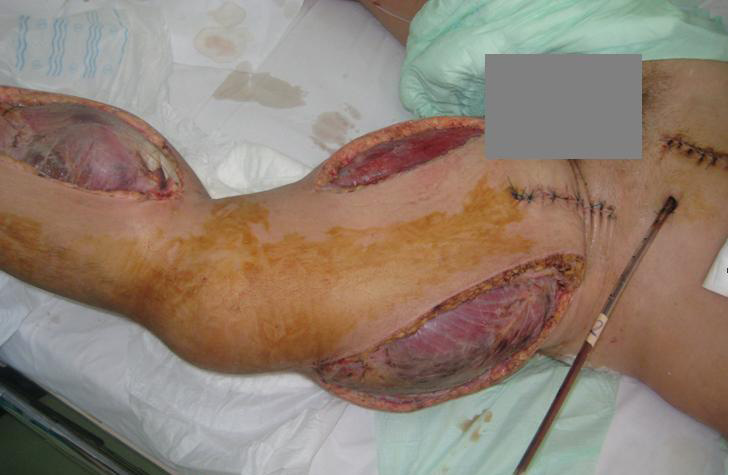
**Extensive crural and femoral fasciotomies**.

A second surgery was performed eight hours after the first one. At his left iliac fossa no significant hematoma was noted. However, there was no pulse over his external iliac artery. After the division of the surrounding tissues it was possible to feel the obstructing foreign body within the common iliac artery. Once vascular control was obtained the artery was opened and the bullet removed (Figure 3 and 4). The embolectomy of the distal arteries was performed using a Fogarty catheter and a significant amount of thrombi was removed (Figure 5). A pulse then returned to his leg. To alleviate developing compartment syndrome, crural and femoral fasciotomy were performed (Figure 6).

Although the leg performed well after the surgery, the postoperative period was complicated by multiorgan failure, which resulted in the death of our patient eighth days after receiving the injury.

## Discussion

As previously stated, an elastic aorta is essential to avoid fatal hemorrhage in patients with a gunshot wound to the aorta. Trimble's [1] collected series gathered from the literature shows bullet embolization is three times more frequent in the lower extremities than in the upper extremities. The forces acting on a migrating embolus to determine the direction of its movement are the force of blood flow, gravity and position of the body. Embolic projectiles that enter the left side of the aortic arch or the abdominal aorta may be expected to be found in a lower extremity. Those that enter the arterial system through the left side of the heart or the right side of the aortic arch may go to either an upper or lower extremity. Emboli on the left side are more frequent, as noted by Garzon and Gleidman [15] and by Keeley [16]. The right and left common iliac arteries arise from the bifurcation at different angles. The left artery is 30° from the midline, more nearly a straight continuation of the aorta than the right iliac which is 45° from the midline. Embolisms to the lower extremities are therefore three times more frequent on the left side than on the right side. The importance of prompt removal of the peripherally located projectile after embolisms is generally stressed in the literature [15], otherwise gangrene may develop. However, the development of gangrene depends more on whether both femoral arteries are occluded than on the length of time itself [2,16]. Due to the absence of a proper diagnostic evaluation at the first surgery we were not able to say if the arterial obstruction occurred in the period between the first and second surgery or was missed the first time. In the case of the possibility of the obstruction occurring between the operations, there are two scenarios to consider. First, the migration of the projectile due to the movement of the spine fragments over the incarcerated bullet. Transportation of our patient from "table to table" may have facilitated detachment of the bullet from his aortic wall and migration to his iliac artery and resulted in a "secondary embolism". If this was the case, than this will be the first such event ever reported in the literature. The second scenario implies the failure to preoperatively and intra-operatively detect the bullet in the iliac artery, and subsequent worsening of ischemia after the first surgery due to the apposition thrombus formation around the already incarcerated bullet in his iliac artery. Regardless of the scenario, there is no doubt that the massive compartment syndrome, developed due to prolonged ischemia, significantly contributed to the lethal outcome in our patient.

## Conclusion

Not all gunshot injuries to the aorta are fatal. If the energy of the projectile diminishes and the aortic wall is elastic enough, the surrounding muscles will prevent exsanguination. The projectile itself can act as an embolus and travel through the vessels. The suspicion for this should rise in all cases when there is a gunshot injury to the abdomen or chest without an exit wound and with no projectile in the area. Failure to recognize this is associated with serious, often irreparable, damage to the patient's health and can even result in a lethal outcome.

## Consent

Written informed consent was obtained from the patient's next of kin for publication of this case report and any accompanying images. A copy of the written consent is available for review by the Editor-in-Chief of this journal.

## Competing interests

The authors declare that they have no competing interests.

## Authors' contributions

JL and AB performed the surgery, and analyzed and interpreted the data. IJV and AT reviewed the literature. All authors were major contributors to the manuscript. All authors read and approved the final manuscript.
